# Circular RNA BIRC6 depletion promotes osteogenic differentiation of periodontal ligament stem cells via the miR-543/PTEN/PI3K/AKT/mTOR signaling pathway in the inflammatory microenvironment

**DOI:** 10.1186/s13287-022-03093-7

**Published:** 2022-08-13

**Authors:** Xinyuan Zhao, Wenjuan Sun, Bing Guo, Li Cui

**Affiliations:** 1 Stomatological Hospital, Southern Medical University, Guangzhou, 510280 China; 2 Department of Stomatology, The Third Affiliated Hospital, Sun Yat-Sen University, Guangzhou, 510630 China; 3 Department of Dentistry, The First Affiliated Hospital, Sun Yat-Sen University, Guangzhou, 510080 China; 4 School of Dentistry, University of California, Los Angeles, Los Angeles, CA 90095 USA

**Keywords:** circBIRC6, Osteogenic differentiation, Periodontal ligament stem cells, Inflammatory microenvironment, miR-543, PTEN

## Abstract

**Background:**

Periodontal ligament stem cells (PDLSCs) are the ideal seed cells for periodontal tissue regeneration. It is well established that persistent inflammation significantly impairs the osteogenic differentiation capability of PDLSCs. Therefore, maintaining PDLSC osteogenic potential under the inflammatory microenvironment is important for treating bone loss in periodontitis. The aim of our study was to explore the potential role of circular RNA BIRC6 (circBIRC6) in regulating osteogenic differentiation of PDLSCs in the inflammatory conditions.

**Methods:**

Alkaline phosphatase staining, Alizarin Red staining, quantitative real-time polymerase chain reaction, western blotting and immunofluorescence staining were used to evaluated the effects of circBIRC6 on the osteogenic differentiation of PDLSCs. RNA pull-down and luciferase assays were performed to explore the interaction between circBIRC6 and miR-543. Then, the downstream signaling pathway affected by circBIRC6/miR-543 axis was further investigated.

**Results:**

The expression level of circBIRC6 was higher in PDLSCs exposed to inflammatory stimulus and in periodontitis tissues compared to the respective controls. Downregulation of circBIRC6 enhanced the osteogenic potential of PDLSCs under the inflammatory conditions, and upregulation of circBIRC6 led to opposite findings. Mechanistically, we found that circBIRC6 modulated PDLSC osteogenic differentiation through sponging miR-543. More importantly, we have demonstrated that circBIRC6/miR-543 axis regulated the mineralization capacity of PDLSCs via PTEN/PI3K/AKT/mTOR signaling pathway in the inflammatory microenvironment.

**Conclusions:**

In summary, the expression of miR-543 is significantly increased following circBIRC6 downregulation, leading to inhibition of PTEN and subsequently activation of PI3K/AKT/mTOR signaling pathway. Therefore, targeting circBIRC6 might represent a potential therapeutic strategy for improving bone loss in periodontitis.

**Supplementary Information:**

The online version contains supplementary material available at 10.1186/s13287-022-03093-7.

## Background

Periodontitis is a chronic, multifactorial inflammatory disease that is characterized by destruction of bone and supporting soft tissues surrounding the tooth [1, 2]. In addition, accumulating evidence indicates that periodontitis is a potential risk factor for a number of systemic conditions [3]. Unfortunately, the currently available periodontal treatments can only retard the progression of periodontitis with minimal tissue regeneration. Therefore, it is urgently needed to explore and develop effective and safe periodontal therapies.

The goal of periodontal therapy is to fully restore the damaged/lost supporting periodontal tissues. The regeneration of periodontitis-related bone loss is a key challenge in clinical dentistry, and enhancing the osteogenic differentiation capacity of seed cells is of great importance for treating periodontitis. Due to the potential to differentiation into osteoblasts, the capability to modulate the immune and inflammatory milieu and lack of ethical issues, periodontal ligament stem cells (PDLSCs) are becoming attractive candidates for regenerating bone defects in the periodontitis microenvironment [4]. However, increasing evidence has demonstrated that the local periodontitis microenvironment significantly impairs the osteogenic potential of PDLSCs [5, 6]. Thus, maintaining the normal biological function of PDLSCs, especially their osteogenic potential, in the harsh inflammatory microenvironment is a major challenge for periodontal tissue repair and regeneration.

Circular RNAs (circRNAs) are a type of evolutionarily conserved, covalently closed non-coding RNAs generated from backsplicing of exons, introns, or both [7, 8]. CircRNAs are highly abundant in eukaryotic transcriptome and are more stable than the linear RNAs due to the characterized loop structure. CircRNAs have been shown to play a critical role in regulating gene and protein expressions by acting as microRNA (miRNA) sponge, or directly translating proteins, or interacting with proteins to affect downstream signaling pathways, etc. [7]. CircRNAs are important for modulating the osteogenic differentiation capacity of PDLSCs. For instance, the expression profile of circRNAs was found to be significantly changed during PDLSC osteogenic differentiation [9]. Ye et al. [10] reported that circFAT1 depletion suppressed the osteogenic differentiation of PDLSCs in vitro and bone formation in vivo, suggesting that circFAT1 might be a potential candidate regulator of PDLSC osteogenesis. Similarly, the expression level of CDR1as was significantly increased during osteogenic differentiation of PDLSCs. Both in vitro and in vivo results revealed that targeting CDR1as markedly inhibited the osteogenic potential of PDLSCs [11]. These findings indicate that circRNAs are actively involving in modulating the osteogenic capacity of PDLSCs, and targeted manipulation of circRNA level might be a novel and promising strategy for bone regeneration under the condition of periodontitis.

Circular RNA BIRC6 (circBIRC6) is a recently discovered circRNA and located on chromosome 2. CircBIRC6 is enriched in human embryonic stem cells (hESCs) and plays an important role in maintaining the pluripotent state of hESCs [12]. The expression level of circBirc6 was found to be deregulated in the myocardial infarction mouse hearts [13]. In addition, abnormal expression of circBIRC6 is actively involved in regulation of tumorigenesis [14, 15]. These currently available findings indicate that circBIRC6 is important for maintaining normal cellular function. Our preliminary study revealed that circBIRC6 level was significantly increased in PDLSCs under the in vitro inflammatory microenvironment, suggesting that its aberrant expression might affect the biological functions of PDLSCs. In this study, we aimed to explore the effects of circBIRC6 on the biological functions of PDLSCs under the inflammatory conditions.

## Materials and methods

### Clinical samples

All clinical samples were obtained from the Stomatological Hospital, Southern Medical University. Written informed consent was obtained from the participants. This study was approved by the Ethics Committee of the Stomatological Hospital, Southern Medical University.

### Cell culture

The PDLSCs were isolated from the periodontal ligament tissues of healthy impacted third molars and then maintained in Dulbecco’s modified Eagle’s medium (DMEM) supplied with 10% fetal bovine serum, 100 U/mL penicillin and 100 μg/mL streptomycin. For the osteogenic differentiation induction, the PDLSCs were cultured with Mesenchymal Stem Cell Osteogenic Differentiation Medium (Cyagen Biosciences, Guangzhou, China). TNF-α (10 ng/mL), IL-1β (10 ng/mL) and *P. gingivalis*-LPS (10 µg/mL) were used to mimic an in vitro inflammatory microenvironment.

### Plasmid construction and cell transfection

The circBIRC6 overexpression plasmid was generated by inserting the circBIRC6 sequence into the pLCDH-ciR vector. The shRNAs targeting circBIRC6 were inserted into the lentiviral vector, and shRNA oligonucleotide sequences were designed by GenePharma (Shanghai, China). Mutant circBIRC6 was constructed by changing the interaction residues between circBIRC6 and miR-543. The miR-543 mimic, miR-543 inhibitor and their respective controls were synthesized by GenePharma. The PTEN overexpression plasmid was constructed by inserting full-length PTEN into the vector. The PTEN shRNA lentiviral particles were purchased from Santa Cruz Biotechnology, Inc. (Santa Cruz, CA, USA). Lentiviruses with circBIRC6 overexpression/inhibition and PTEN overexpression were produced as previously described [16]. The PDLSCs were infected with lentiviruses and/or transfected with miRNA mimics/inhibitors using Lipofectamine 3000 (Invitrogen, Carlsbad, CA, USA) according to the manufacturer’s instructions.

### Quantitative real-time polymerase chain reaction analysis

Total RNA was extracted with the Quick-RNA™ kit (Zymo Research Corp, Irvine, CA, USA). The SuperScript™ III First-Strand Synthesis SuperMix (Invitrogen) was used to synthesize first-strand cDNA. The amplification of cDNA was performed with Light Cycler 480@ SYBR Green I MasterMix (Roche, Applied Science, Indianapolis, IN, USA) on the CFX96 Real-Time PCR detection system (Bio-Rad, Hercules, CA, USA). GAPDH and U6 were used as the internal controls, and the relative gene expression levels were determined by the 2^−ΔΔCT^ method. The circBIRC6 primers were designed and provided by GenePharma. The primer sequences used in this study are listed in Additional file 1: Table S1.

### Western blotting

Followed by separation with 4–20% tris-glycine gels, the proteins were transferred onto a PVDF membrane using a Trans-Blot Turbo system (Bio-Rad). After soaking the membrane in 5% nonfat milk for 1 h at room temperature, the primary antibodies and HRP-linked secondary antibodies (Proteintech, Chicago, IL, USA) were used to detect the targeted proteins. The primary antibodies for BMP2, OPN, PTEN, PI3K, AKT, p-AKT, mTOR and p-mTOR were from Proteintech. The p-PI3K primary antibody was from Abcam. The primary antibodies against OCN and BSP were from Santa Cruz Biotechnology and Thermo Fisher Scientific, respectively.

### Immunofluorescence staining

Briefly, the cells were fixed with paraformaldehyde for 10 min and then permeated with 0.1% Triton X-100 for 10 min. After washing three times with PBS, the samples were blocked with immunostaining blocking buffer (Beyotime, Shanghai, China) for 1 h at room temperature. Then, the cells were incubated with primary antibodies against RUNX2 (Proteintech) and COL1A1 (Proteintech) at 4 °C overnight, and subsequently with a fluorescence-conjugated secondary antibody at room temperature for 1 h. Images were taken with an inverted fluorescence microscope (Leica Microsystems, Wetzlar, Germany).

### Alkaline phosphatase (ALP) staining

The cells were fixed with 4% paraformaldehyde for 10 min after 10 days induction of osteogenic differentiation. Then, the samples were incubated in 0.1 M Tris buffer (pH 9.3) containing 0.25% naphthol AS-BI phosphate (Sigma-Aldrich, Saint Louis, MO, USA) and 0.75% Fast Blue BB (Sigma-Aldrich) at 37 °C.

### Alizarin Red S (ARS) staining

Briefly, after three-week osteogenic differentiation induction, the fixed cells were stained with Alizarin Red S solution (Cyagen Biosciences) for 20 min at room temperature.

### MTT assay

The cells were incubated with MTT solution (Sigma-Aldrich) for 4 h at 37 °C. Then, dimethyl sulfoxide was used to dissolve the sediment. The absorbance was determined with a Synergy HT microplate reader (BioTek Instruments, Winooski, VT, USA) at 570 nm wavelength.

### EdU (5-ethynyl-2’-deoxyuridine) assay

The cells were incubated with 10 μM EdU for at 37 °C for 2 h, and then, the fixation and permeabilization of the samples were performed with 3.7% formaldehyde and 0.1% Triton X-100, respectively. The Click-iT™ reaction cocktail (Invitrogen) and Hoechst 33342 were used to stain the cells. Images were taken with an inverted fluorescence microscope (Leica Microsystems).

### Flow cytometry

The harvested cells were fixed with ice-cold ethanol overnight. After removing the fixative, the samples were stained with propidium iodide and incubated in dark for 20 min. The cell cycle distribution was detected using DxFLEX Flow Cytometer (Beckman Coulter, Brea, CA, USA).

### RNA immunoprecipitation (RIP)

The RIP assay was performed using a Magna RIP Kit (Millipore, Billerica, MA, USA) according to the manufacturer’s instructions. The AGO2 antibody (Abcam) and the negative control IgG antibody were used for the RIP experiments.

### Pull-down assay

Briefly, the biotinylated circBIRC6 probe and oligo probe were incubated with Streptavidin-Dyna beads M-280 (Invitrogen). Then, the cell lysates were incubated with beads at 4 °C overnight, and the beads were subsequently washed. The RNA was extracted and evaluated by qRT-PCR.

### Luciferase reporter assay

The circBIRC6 wt, circBIRC6 mut, miR-543 mimic, miR-543 inhibitor and miRNA negative controls were transfected into PDLSCs alone or in combination using the Lipofectamine 3000 transfection reagent (Invitrogen). Following 24 h of incubation, the luciferase reporter assay kit (Promega, Madison, WI, USA) was used to determine the relative luciferase activities.

### Statistical analysis

All statistical analyses were performed with the GraphPad Prism 9.0 (GraphPad Software, San Diego, CA, USA). Student’s t test and one-way ANOVA were used to compare the difference between groups. The correlation between circBIRC6 and miR-543 in periodontitis tissues was evaluated with the Pearson correlation analysis. *P* values less than 0.05 were considered as statistically significant.

## Results

### The osteogenic differentiation of PDLSCs is impaired in the inflammatory microenvironment

**Fig. 1:**
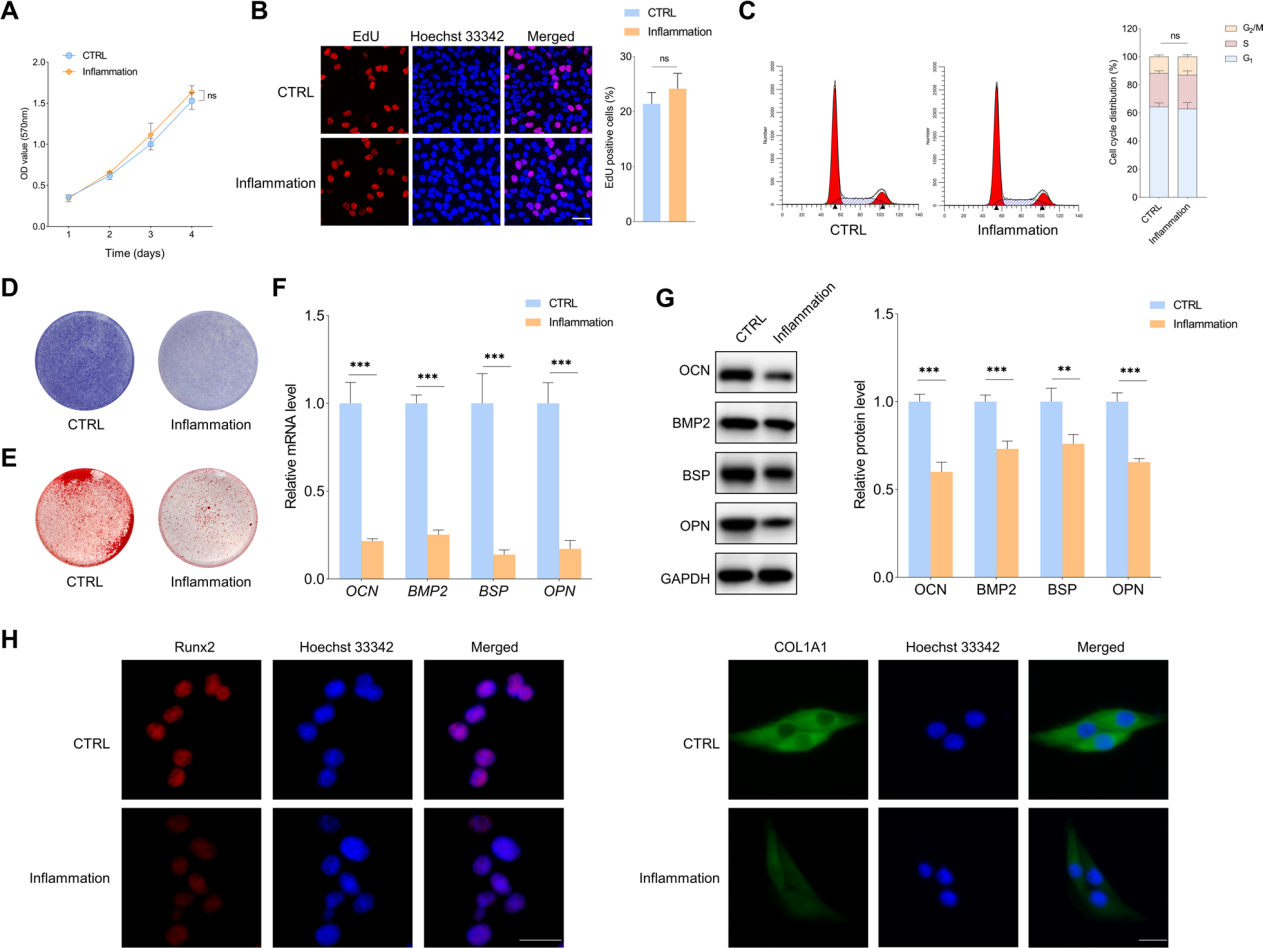
The effects of inflammatory microenvironment on the proliferation and osteogenic differentiation of PDLSCs. **A** MTT assay of PDLSCs in the normal condition and inflammatory environment. **B** Representative images of the EdU assay in PDLSCs with the indicated treatments. Scale bar: 50 μm. **C** The flow cytometry revealed the cell cycle distribution of PDLSCs with the indicated treatments. **D** ALP staining of PDLSCs following a 10-day osteogenic differentiation induction in the normal and inflammatory microenvironment. **E** ARS staining of PDLSCs following a three-week osteogenic differentiation induction with the indicated treatments. **F**, **G** qRT-PCR and immunoblot of osteogenic differentiation markers in PDLSCs with indicated treatments. **H** Immunofluorescence staining of RUNX2 and COL1A1 in PDLSCs in the normal and inflammatory conditions. Scale bar: 20 μm. Data are presented as the mean ± SD (ns: not significant, **P < 0.01, ***P < 0.001)

The in vitro inflammatory microenvironment was mimicked by adding TNF-α, IL-1β and *P. gingivalis*-LPS to the culture medium. Then, the effects of inflammatory conditions on the proliferation and osteogenic differentiation of PDLSCs were evaluated. The MTT assay showed that the OD values changed little between the PDLSCs under the normal condition and those exposed to inflammation (Fig. 1A). Similarly, no significant alteration was found for the percentage of EdU positive cells and cell cycle distribution between the control group and the inflammation group (Fig. 1B, C), indicating that the inflammatory microenvironment has little effects on the proliferation of PDLSCs. For the osteogenic differentiation, the ALP staining assay showed that the staining intensity was lower in the inflammation group compared to the control group (Fig. 1D). The ARS staining revealed that cells exposure to the inflammatory conditions formed significantly less mineralized nodules than those grown in the normal growth microenvironment (Fig. 1E). In addition, the qRT-PCR and WB demonstrated that the levels of osteogenic differentiation markers OCN, BMP2, BSP and OPN were markedly lower in the inflammation group than in the control group (Fig. 1F, G). Similarly, the immunofluorescence staining showed that lower positive immunofluorescence signals for RUNX2 and COL1A1 were detected in the inflammation group (Fig. 1H). The above results suggest that the in vitro inflammatory microenvironment dramatically reduces the osteogenic differentiation of PDLSCs.

### *CircBIRC6 depletion promotes osteogenic differentiation of PDLSCs in the inflammatory microenvironment, and *vice versa

**Fig. 2:**
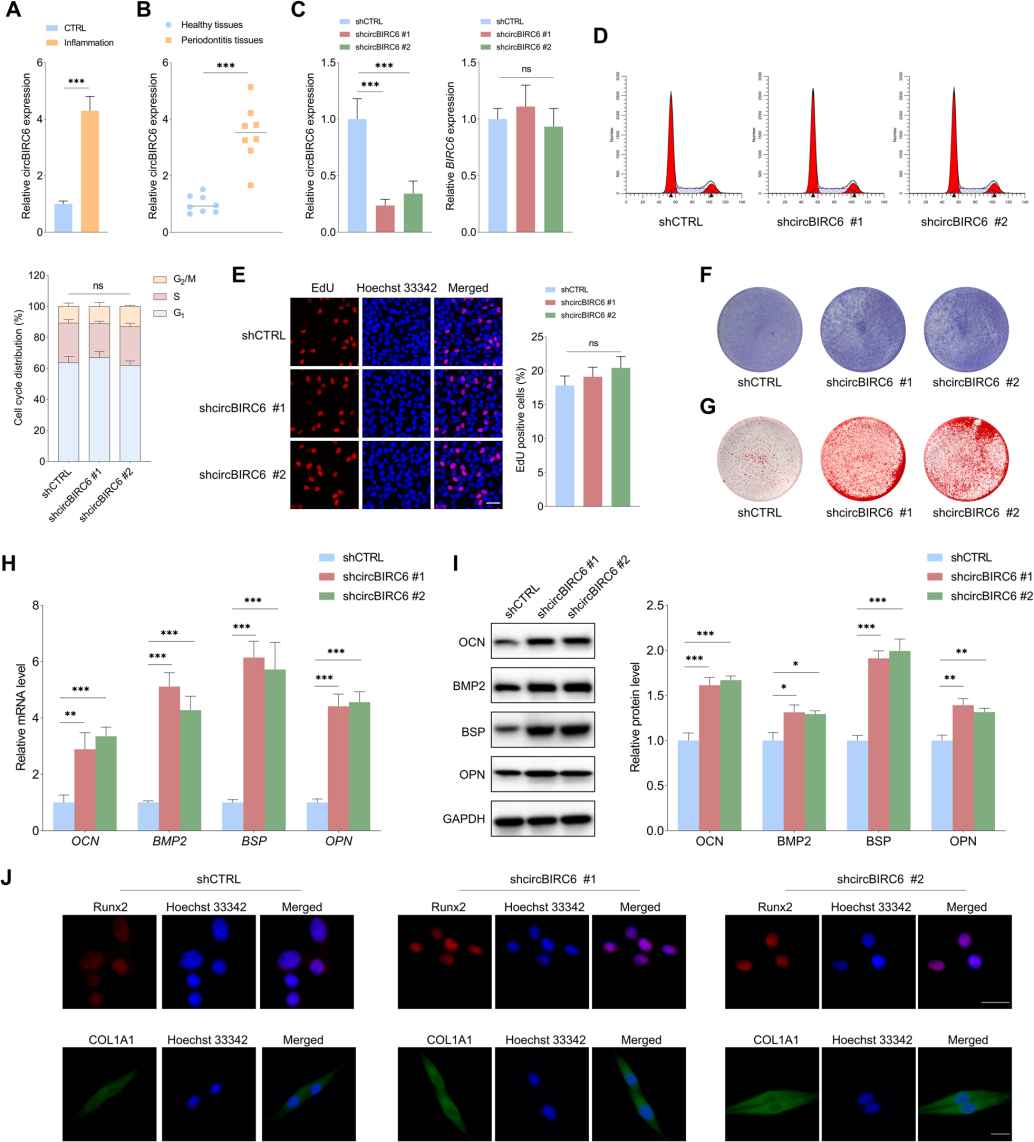
CircBIRC6 depletion promoted the osteogenic differentiation of PDLSCs under the inflammatory microenvironment. **A** CircBIRC6 level was significantly higher in PDLSCs exposed to inflammatory stimulus. **B** The expression level of circBIRC6 was markedly higher in periodontitis tissues compared to normal tissues. **C** Establishment of circBIRC6-stable knockdown PDLSCs, and the relative levels of circBIRC6 and linear *BIRC6* were detected with qRT-PCR. **D**, **E** Flow cytometry and EdU assays showed that downregulation of circBIRC6 had little effect on the proliferative capacity of PDLSCs under the inflammatory conditions. Scale bar: 50 μm. **F** The ALP staining intensity was higher in the circBIRC6 knockdown groups compared to the control group. **G** ARS assay of the mineralized nodules formation capacity of PDLSCs with the indicated modifications. **H–J** qRT-PCR, immunoblot and immunofluorescence staining of osteogenic differentiation markers in PDLSCs with the indicated modifications. Scale bar: 20 μm. Data are presented as the mean ± SD (ns: not significant, *P < 0.05, **P < 0.01, ***P < 0.001)

**Fig. 3:**
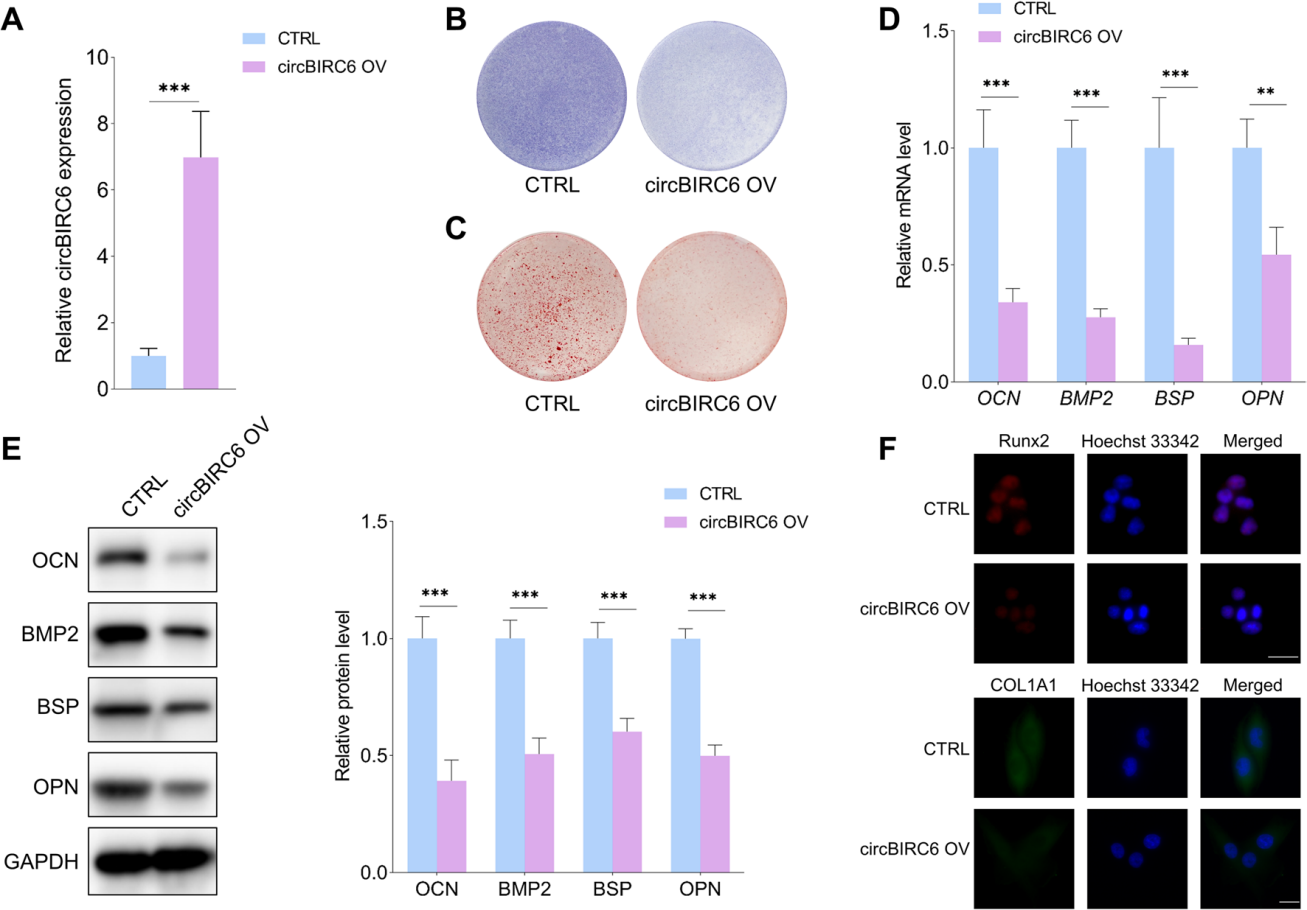
Overexpression of circBIRC6 suppressed the osteogenic potential of PDLSCs in the inflammatory conditions. **A** Establishment of circBIRC6-overexpressing PDLSCs. **B** The ALP staining intensity of PDLSCs in the circBIRC6-overexpressing PDLSCs and control PDLSCs under the inflammatory microenvironment. **C** ARS staining of mineralized nodules formed by PDLSCs with indicated modifications. **D–F** qRT-PCR, immunoblot and immunofluorescence staining of osteogenic differentiation markers in PDLSCs with indicated modifications. Scale bar: 20 μm. Data are presented as the mean ± SD (**P < 0.01, ***P < 0.001)

As shown in Fig. 2A, the expression level of circBIRC6 was significantly increased in the inflammatory microenvironment. In addition, circBIRC6 was overexpressed in periodontitis tissues compared to healthy periodontal tissues (Fig. 2B). The aberrant expression of circBIRC6 both in vitro and in vivo periodontitis models indicates that it might play an important role in regulating the biological functions of PDLSCs under the inflammatory conditions. The shcircBIRC6 significantly reduced the expression level of circBIRC6 in PDLSCs, whereas had little effect on the linear *BIRC6* expression (Fig. 2C). Then, the effects of circBIRC6 downregulation on the proliferation and osteogenic differentiation of PDLSCs under inflammatory conditions were evaluated. The flow cytometry and EdU assays showed that circBIRC6 depletion did not affect the proliferative capacity of PDLSCs (Fig. 2D, E). Interestingly, the ALP staining intensity and the mineralized nodule formation capacity were significantly higher in the shcircBIRC6 groups than in the control group (Fig. 2F, G). The expression levels of OCN, BMP2, BSP and OPN were found consistently upregulated at both mRNA and protein levels in the shcircBIRC6 groups compared to the control group (Fig. 2H, I). Similarly, higher RUNX2 and COL1A1 immunofluorescence staining were observed in the circBIRC6 depletion groups (Fig. 2J). These results support that circBIRC6 might act as a negative regulator of PDLSC osteogenic differentiation in the inflammatory microenvironment. Then, circBIRC6 was overexpressed to explore its effects on the osteogenic differentiation of PDLSCs (Fig. 3A). The ALP and ARS staining assays both demonstrated that circBIRC6 overexpression significantly decreased the osteogenic potential of PDLSCs under the inflammatory conditions (Fig. 3B, C). In addition, as shown in Fig. 3D, E, and F, the levels of osteogenic differentiation markers were markedly decreased in the circBIRC6-overexpressing group compared to the control group.

### CircBIRC6 directly binds to miR-543 and acts as a competitive endogenous RNA

**Fig. 4:**
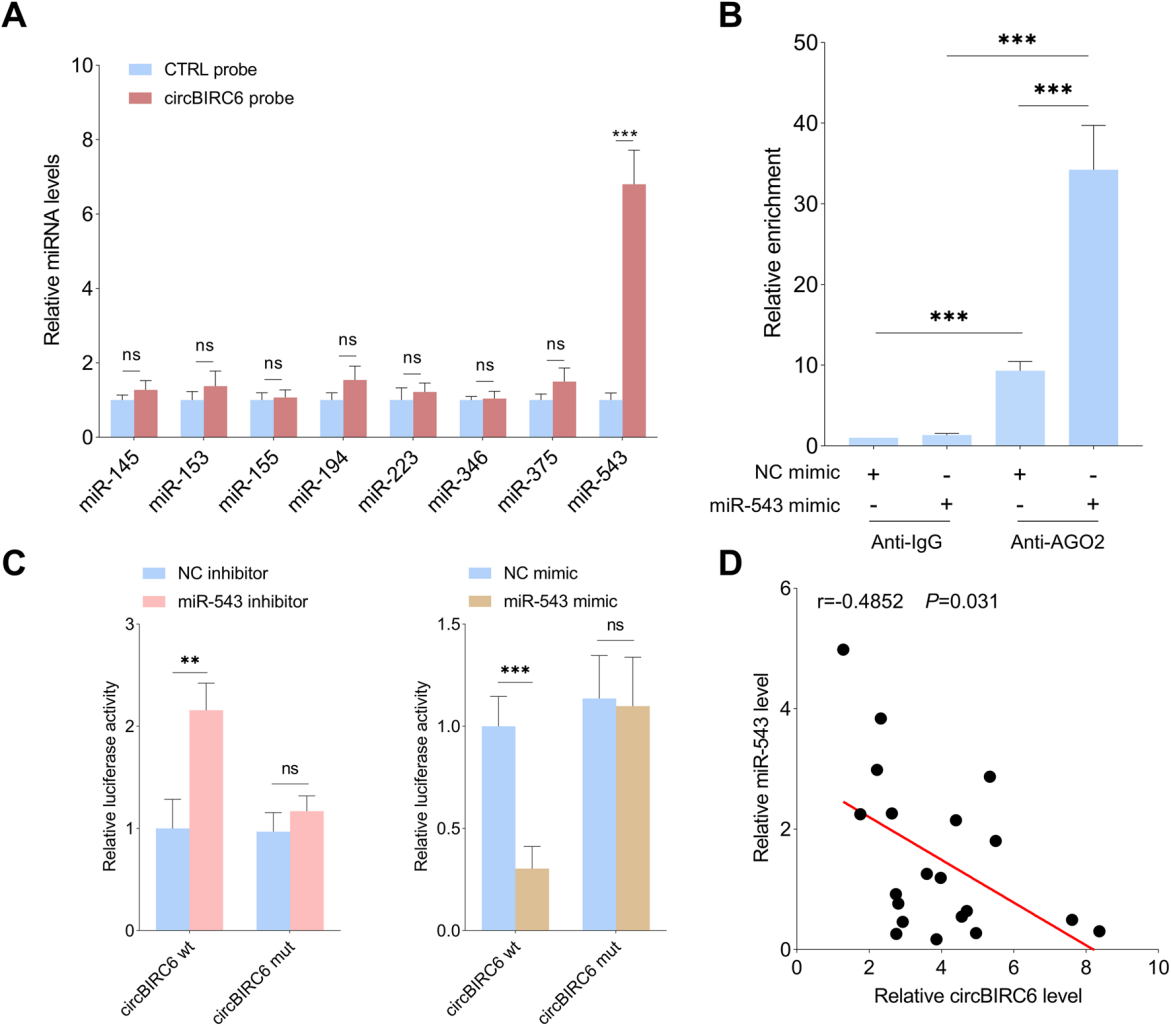
CircBIRC6 acted as an efficient miR-543 sponge in PDLSCs. **A** qRT-PCR was performed to evaluate the relative expression levels of potential target miRNAs pulled down by the circBIRC6 probe or oligo probe. **B** RIP assay was conducted with anti-AGO2 or anti-IgG in PDLSCs after transfection with the miR-543 mimic or NC mimic, and qRT-PCR was used to detect the relative enrichment of circBIRC6. **C** Relative luciferase activities in PDLSCs co-transfected with circBIRC6 wt or circBIRC6 mut and the miR-543 mimic, miR-543 inhibitor or respective negative controls. **D** The correlation between circBIRC6 and miR-543 in periodontitis tissues was evaluated. Data are presented as the mean ± SD (ns: not significant, **P < 0.01, ***P < 0.001)

It is well established that circRNAs exert their biological functions through the miRNA sponge mechanism. We first screened the potential miRNAs that might bind the circBIRC6 using the online tool (https://circinteractome.nia.nih.gov/). Then, only those that have been reported to be associated with osteogenic differentiation were chosen for further evaluation. Among all tested miRNAs, miR-543 was the only miRNA that was copiously pulled down by circBIRC6 probe in PDLSCs (Fig. 4A). The RNA immunoprecipitation (RIP) assay showed that circBIRC6 was pulled down in dramatically higher quantity by the anti-AGO2 than anti-IgG. In addition, in the anti-AGO2 pull-down cells, the level of circBIRC6 was significantly higher in the miR-543 mimic group compared to NC mimic group (Fig. 4B). Based on the complementary sequences, the mutant circBIRC6 was constructed by changing the binding sites between circBIRC6 and miR-543. The luciferase reporter assay showed that miR-543 inhibitor promoted circBIRC6 wt reporter activity, while miR-543 mimic suppressed its activity. The luciferase activity of the circBIRC6 mut was not significantly altered following the miR-543 overexpression or inhibition (Fig. 4C). These findings indicate that circBIRC6 directly binds to miR-543. The correlation between circBIRC6 and miR-543 was further evaluated in periodontitis tissues. The results showed that miR-543 expression was negatively associated with circBIRC6 expression in periodontitis tissues (Fig. 4D).

### MiR-543 promotes osteogenic differentiation of PDLSCs under the inflammatory microenvironment

**Fig. 5:**
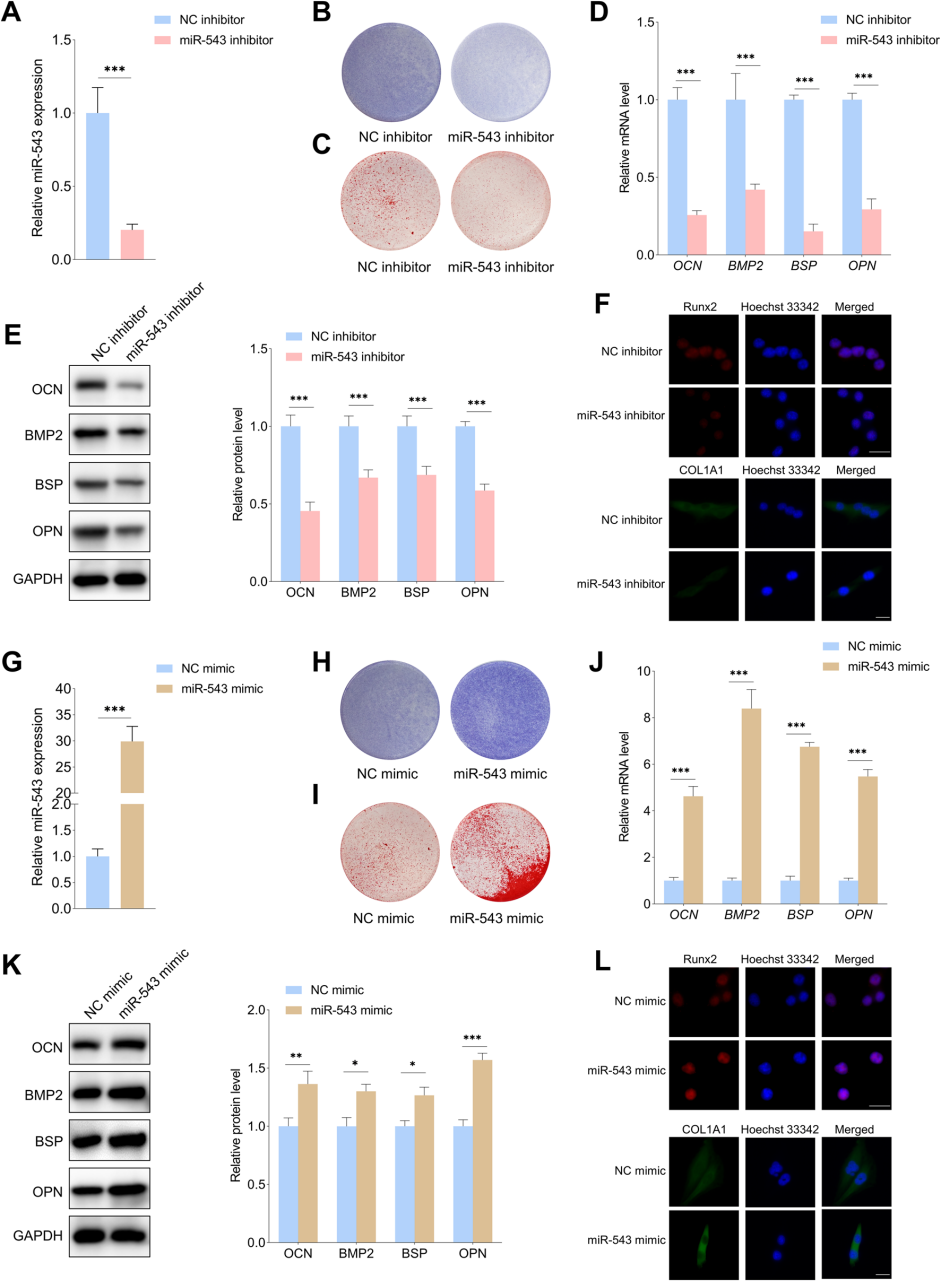
MiR-543 promoted the osteogenic differentiation of PDLSCs under the inflammatory microenvironment. **A** The expression of miR-543 in PDLSCs was analyzed by qRT-PCR after transfection with miR-543 inhibitor and NC inhibitor. **B–F** The osteogenic differentiation capability of PDLSCs transfected with miR-543 inhibitor in the inflammatory conditions was evaluated by ALP staining, ARS staining, qRT-PCR, WB and immunofluorescence staining. **G** The miR-543 level was detected with qRT-PCR after transfection with miR-543 mimic and NC mimic. **H–L** The effects of miR-543 mimic on the osteogenic differentiation of PDLSCs under the inflammatory microenvironment were evaluated by ALP staining, ARS staining, qRT-PCR, WB and immunofluorescence staining. Data are presented as the mean ± SD (*P < 0.05, **P < 0.01, ***P < 0.001). Scale bar: 20 μm (**F** and **L**)

The effects of miR-543 on the osteogenic differentiation of PDLSCs under the inflammatory conditions were then evaluated. The results showed that the ALP staining intensity and the mineralized nodule formation capacity were markedly lower in the miR-543 inhibitor group than in the NC inhibitor group (Fig. 5A, B, and C). In addition, miR-543 inhibitor suppressed the expression of osteogenic differentiation markers in the inflammatory microenvironment (Fig. 5D, E, and F). As shown in Fig. 5G–L, overexpression of miR-543 promoted the osteogenic differentiation of PDLSCs in the inflammatory conditions. The above results suggest that miR-543 positively regulates the osteogenic potential of PDLSCs in the inflammatory conditions.

### CircBIRC6 regulates osteogenic differentiation of PDLSCs by sponging miR-543 in the inflammatory environment

**Fig. 6:**
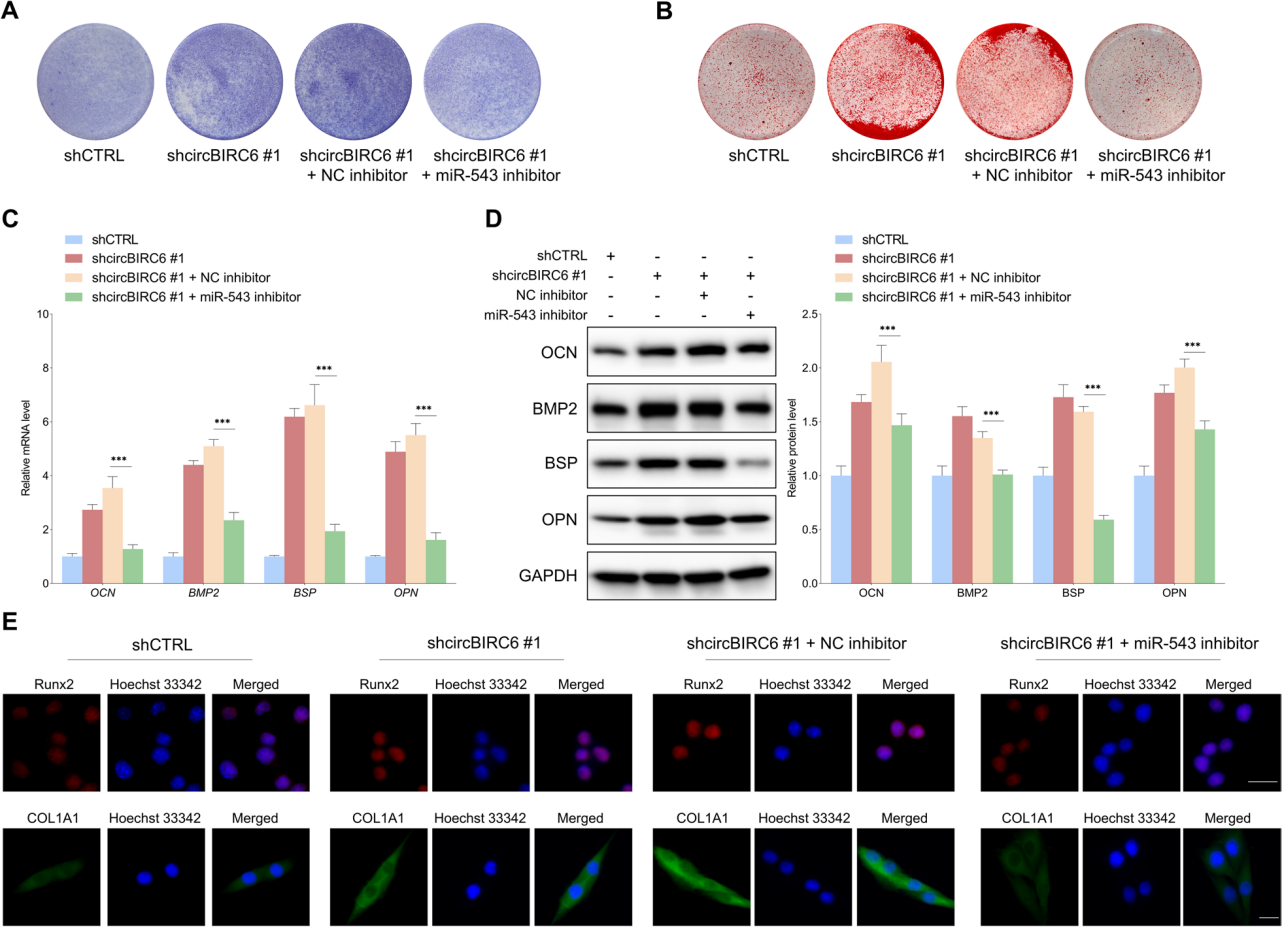
MiR-543 inhibitor suppressed the osteogenic differentiation capacity of PDLSCs enhanced by circBIRC6 downregulation under the inflammatory microenvironment. **A** ALP staining of PDLSCs with indicated modifications. **B** ARS staining of mineralized nodules formed by PDLSCs with indicated treatments. **C–E** qRT-PCR, WB and immunofluorescence staining were performed to detect the changes in osteogenic differentiation makers in PDLSCs with indicated modifications. Scale bar: 20 μm. Data are presented as the mean ± SD (***P < 0.001)

**Fig. 7:**
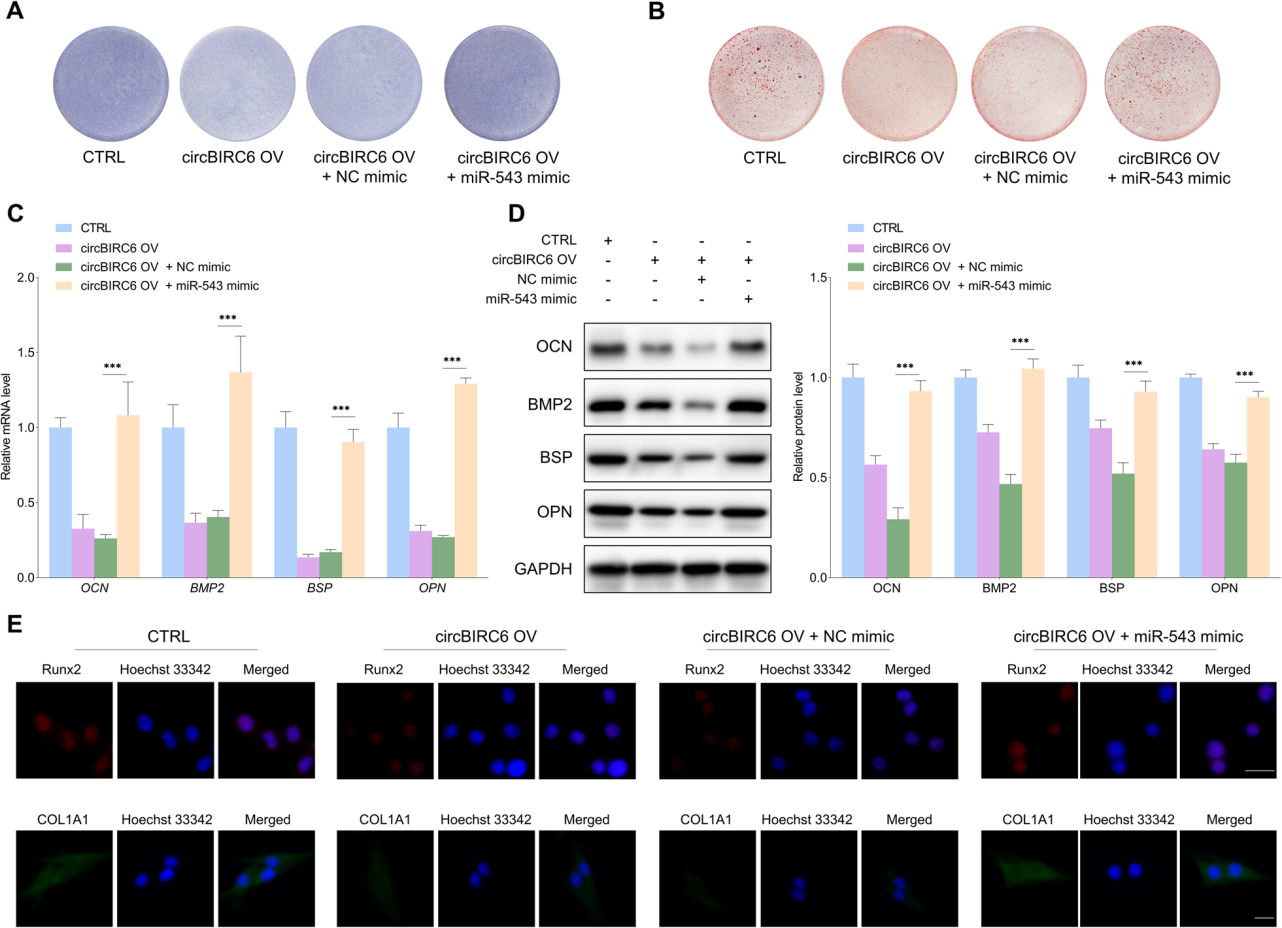
MiR-543 mimic rescued PDLSC osteogenic differentiation capacity inhibited by circBIRC6 overexpression under the inflammatory microenvironment. **A** The ALP staining intensity of PDLSCs with indicated treatments. **B** The mineralized nodules formation capacity of PDLSCs with indicated modifications was examined with ARS staining. **C–E** qRT-PCR, WB and immunofluorescence staining were performed to evaluated the alterations in osteogenic differentiation makers in PDLSCs with indicated treatments. Scale bar: 20 μm. Data are presented as the mean ± SD (***P < 0.001)

The rescue experiments were performed to verify whether miR-543 was a functional downstream target of circBIRC6. The ALP and ARS staining assays showed that downregulation of circBIRC6 increased the ALP staining intensity and promoted mineralized nodule formation, and this effect was partially suppressed by miR-543 inhibitor (Fig. 6A, B). Similarly, the increased levels of osteogenic differentiation markers induced by shcircBIRC6 were reversed by miR-543 inhibitor (Fig. 6C, D, and E). We then further investigated whether miR-543 overexpression reversed the suppressive effects of circBIRC6 upregulation in osteogenic differentiation. As shown in Fig. 7A–E, the inhibitory effects of circBIRC6 overexpression on osteogenic differentiation of PDLSCs were antagonized by upregulation of miR-543. These results strongly support that miR-543 is functional downstream target of circBIRC6 for regulating osteogenic differentiation under the inflammatory conditions.

### CircBIRC6/miR-543 axis regulates PDLSC osteogenic differentiation in the inflammatory conditions through PTEN/PI3K/AKT/mTOR signaling pathway

**Fig. 8:**
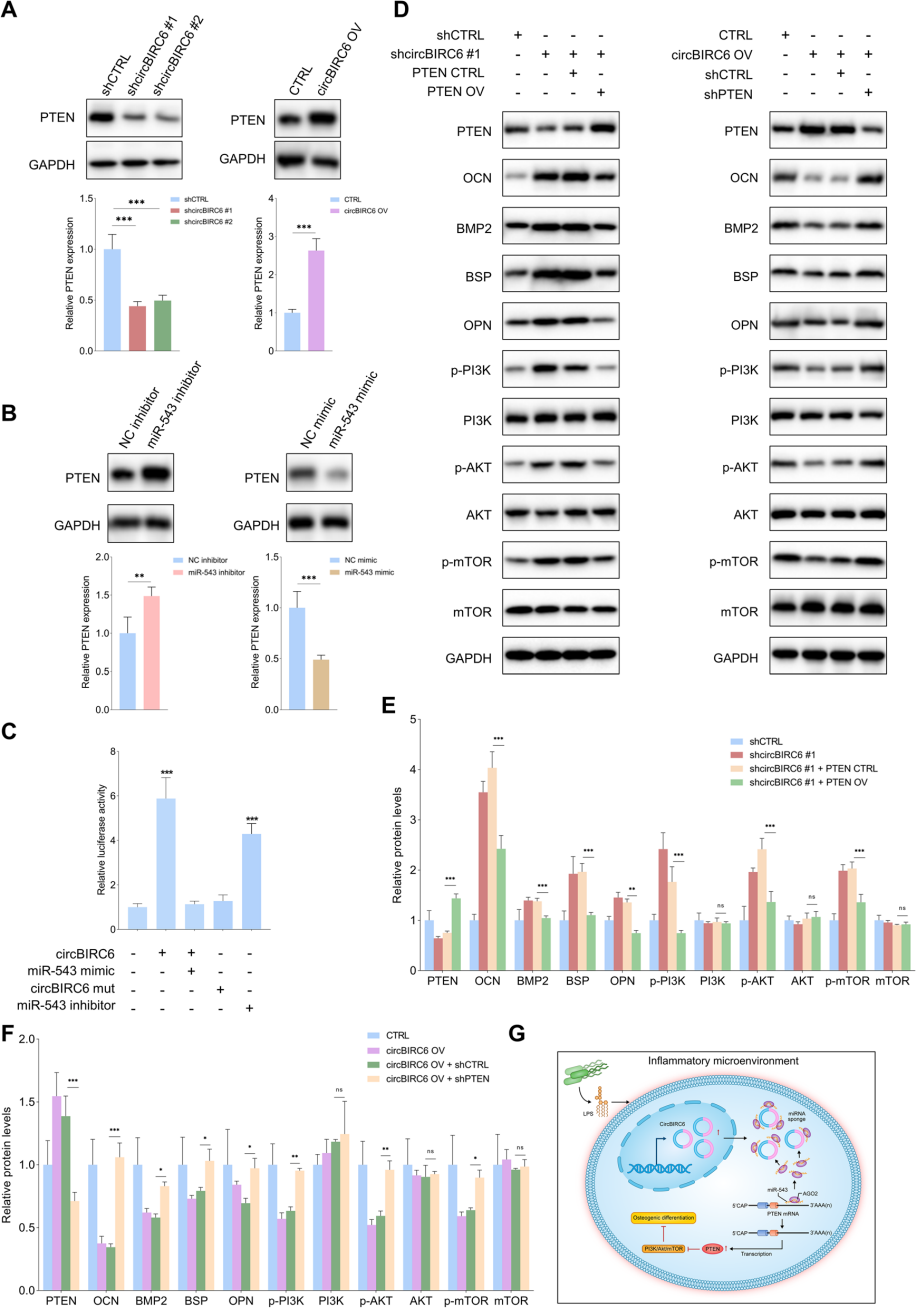
CircBIRC6/miR-543 axis regulated the osteogenic potential of PDLSCs via PTEN/PI3K/AKT/mTOR signaling pathway. **A**, **B** WB assay of PTEN expression level in PDLSCs with indicated modifications. **C** The relative luciferase activities of PTEN promoter in PDLSCs transfected with circBIRC6 wt, circBIRC6 mut. miR-543 mimic and miR-543 inhibitor alone or in combination. **D–F** WB assays of PTEN, osteogenic differentiation markers, p-PI3K, PI3K, p-AKT, AKT, p-mTOR and mTOR in PDLSCs with indicated treatments. **G** Schematic illustration of the circBIRC6/miR-543/PTEN/PI3K-AKT-mTOR axis in regulating the PDLSC osteogenic differentiation under the inflammatory microenvironment. Data are presented as the mean ± SD (ns: not significant, *P < 0.05, **P < 0.01, ***P < 0.001)

To further investigate the downstream genes of miR-543 in PDLSCs, we first screened the currently available validated targets. Based on the results of miRWalk (http://mirwalk.umm.uni-heidelberg.de/) and miRTarBase (http://miRTarBase.cuhk), PTEN was selected as the potential downstream target of further investigation. The WB results showed that shcircBIRC6 suppressed the expression of PTEN, and PTEN level was increased when circBIRC6 was overexpressed (Fig. 8A). Not surprisingly, miR-543 mimic downregulated PTEN expression, and miR-543 inhibitor promoted its expression (Fig. 8B). To further verify the potential role of circBIRC6 in regulating PTEN expression, circBIRC6, circBIRC6 mut, miR-543 mimic and miR-543 inhibitor were transfected into PDLSCs individually or in combination. The results showed that only circBIRC6 and miR-543 inhibitor enhanced the reporter activity of PTEN (Fig. 8C), indicating that circBIRC6/miR-543 axis might be an upstream regulator of PTEN. As shown in Fig. 8D, E, and F, overexpression of PTEN suppressed the circBIRC6 knockdown-mediated increased osteogenic potential of PDLSCs, and PTEN downregulation partially reversed the suppressive effects of circBIRC6 upregulation in osteogenic differentiation. PTEN is a well-known upstream regulator of PI3K/AKT/mTOR, which plays an essential role in maintaining the osteogenic differentiation capability of PDLSCs. Our WB results showed that shcircBIRC6 promoted the expression levels of p-PI3K, p-AKT and p-mTOR, and this effect was counteracted by PTEN overexpression. Similarly, circBIRC6 upregulation reduced the expression levels of p-PI3K, p-AKT and p-mTOR, and downregulation of PTEN rescued their expression. Figure 8G illustrates the potential molecular mechanisms for circBIRC6 in regulating PDLSC osteogenic differentiation under the inflammatory microenvironment.

## Discussion

It is well established that the chronic inflammatory microenvironment attenuates the osteogenic differentiation capacity of MSCs and negatively affects bone repair and regeneration [17, 18]. Alveolar bone defect is one of the common characteristics of periodontitis, and thus, successful bone regeneration under the inflammatory conditions is the key challenge for conquering periodontitis. PDLSCs are the most important seed cells for periodontal regeneration. Therefore, it is of great importance to investigate how to enhance the defective osteogenic differentiation capability of PDLSCs in the inflammatory microenvironment. In this study, our results have clearly demonstrated that circBIRC6 level is increased in PDLSCs exposed to inflammatory stimulus and in periodontitis tissues. Knockdown of circBIRC6 promotes the osteogenic differentiation of PDLSCs under the inflammatory conditions, and opposite findings are observed when circBIRC6 is overexpressed. Mechanistically, circBIRC6 regulates osteogenic differentiation of PDLSCs by sponging miR-543 in the inflammatory environment. More importantly, we have evidenced that circBIRC6/miR-543 axis modulates the mineralization capacity of PDLSCs through PTEN/PI3K/AKT/mTOR signaling pathway.

Recent evidence has demonstrated that circRNAs play an important role in regulating the osteogenic differentiation of MSCs. For instance, circSIPA1L1 was found to promote the osteogenic potential of stem cells from apical papilla by modulating the miR-204-5p/ALPL axis [19]. Similarly, circRNA CDR1as induced the osteogenic differentiation of PDLSCs by sponging miR-7, which further enhanced GDF5 expression and subsequently activated pSmad1/5/8 and p38 MAPK pathway [11]. However, whether circRNAs regulate the osteogenic differentiation of PDLSCs under the inflammatory microenvironment is poorly known. Aberrant expression of circBIRC6 has been reported to be involved in many biological processes such as stem cell pluripotency, myocardial infarction and tumorigenesis [12–14]. To the best of our knowledge, this is the first study showing the downregulation of circBIRC6 enhances the osteogenic differentiation capacity of PDLSCs exposed to the inflammatory conditions, which might provide a novel strategy for promoting bone regeneration in the harsh microenvironment.

Then, we demonstrated that miR-543 was a functional downstream target of circBIRC6 for regulating the osteogenic differentiation of PDLSCs in the inflammatory microenvironment. Consistent with our findings, downregulation of miR-543 suppressed the osteogenic potential of human dental pulp stem cells (DPSCs), and opposite findings were observed when miR-543 was overexpressed [20], indicating that the miR-543 was a positive regulator of DPSC osteogenic differentiation. Similarly, under the normal condition, the expression level of miR-543 was increased during the osteogenic differentiation of PDLSCs. In addition, miR-543 upregulation promoted the osteogenesis of PDLSCs, and vice versa [21]. We speculate that miR-543 is reduced in the inflammatory microenvironment due to the sponging effects of circBIRC6, leading to the defective osteogenic differentiation capability of PDLSCs. In addition to miR-543, whether circBIRC6 sponges other miRNAs for regulating osteogenic differentiation of PDLSCs needs further investigation.

Among the validated downstream targets of miR-543, PTEN is an important regulator for controlling MSC osteogenic differentiation. For instance, PTEN upregulation suppressed the BMP-9 mediated osteogenic potential in MSCs through inhibiting the expression of Wnt10b [22]. Loss of PTEN in mouse osteoblasts promoted the osteogenic differentiation and reduced apoptosis in vitro and in vivo, and the enhancing effects were mediated by activating the AKT [23]. PTEN is well-known negative regulator of PI3K/AKT/mTOR signaling pathway, which is crucial for controlling multiple biological processes including MSC osteogenic differentiation [24]. Therefore, following downregulation of circBIRC6, the sponging effects of circBIRC6 on miR-543 are suppressed, leading to the release of miR-543 in the cytosol. The accumulation of miR-543 inhibits the expression of PTEN, which further results in the activation of PI3K/AKT/mTOR signaling pathway. The activated PI3K/AKT/mTOR pathway promotes the osteogenic differentiation of PDLSCs under the inflammatory conditions.

## Conclusions

Under the inflammatory conditions, downregulation of circBIRC6 promotes the osteogenic differentiation of PDLSCs by modulating miR-543/ PTEN/PI3K/AKT/mTOR signaling pathway. These findings have important implications for the design and implementation of circBIRC6 targeting strategies to treat bone loss in periodontitis.

## Supplementary Information

**Additional file 1**. Sequences of primers and oligos.

## Data Availability

The datasets used and/or analyzed during the current study are available from the corresponding author on reasonable request.

## Abbreviations

ALP: Alkaline phosphatase
ARS: Alizarin Red S
circBIRC6: Circular RNA BIRC6
hESCs: Human embryonic stem cells
mTOR: Mammalian target of rapamycin
MSCs: Mesenchymal stem cells
PDLSCs: Periodontal ligament stem cells
PI3K: Phosphatidylinositol 3-kinase
PTEN: Phosphatase and tensin homolog
