# Supplementary material for: Circular RNA BIRC6 depletion promotes osteogenic differentiation of periodontal ligament stem cells via the miR-543/PTEN/PI3K/AKT/mTOR signaling pathway in the inflammatory microenvironment
Source: Stem Cell Res Ther. 2022 Aug 13;13:417. doi: 10.1186/s13287-022-03093-7 (PMC9375426; doi:10.1186/s13287-022-03093-7)
Supplement: Supplementary file 1 — Additional file 1. Sequences of primers and oligos. [file 13287_2022_3093_MOESM1_ESM.docx]

**Additional file 1: Table S1** Sequences of primers and oligos

| **Gene** | **Sequence (5′-3′)** |
| --- | --- |
| OCN | F: AGCAAAGGTGCAGCCTTTGT |
|  | R: GCGCCTGGGTCTCTTCACT |
| BMP2 | F: CACTGTGCGCAGCTTCC |
|  | R: CCTCCGTGGGGATAGAACTT |
| BSP | F: GCAGTAGTGACTCATCCGAAGAA |
|  | R: GCCTCAGAGTCTTCATCTTCATTC |
| OPN | F: CTCCATTGACTCGAACGACTC |
|  | R: CAGGTCTGCGAAACTTCTTAGAT |
| BIRC6 | F: CAGCAGCTCTTATCAGCATGT |
|  | R: AACTGTGGCCCACTTAGCAAC |
| miR-543 | F: CAGTGCTAAAACATTCGCGG |
| miR-145 | F: CCTTGTCCTCACGGTCCAGT |
| miR-153 | F: ACACTCCAGCTGGGTTGCATAGTCACAAA |
| miR-155 | F: TTAATGCTAATCGTGATAGGGGT |
| miR-194 | F: GCGGCGGTGTAACAGCAACTCC |
| miR-223 | F: TGTCAGTTTGTCAAATACCCCA |
| miR-346 | F: GGGTGTCTGCCCGCATGCCT |
| miR-375 | F: AGCCGTTTGTTCGTTCGGCT |
| GAPDH | F: TGCACCACCAACTGCTTAGC |
|  | R: GGCATGGACTGTGGTCATGAG |
| U6 | F: CTCGCTTCGGCAGCACATATACT |
|  | R: ACGCTTCACGAATTTGCGTGTC |
